# A broad-range polymerase chain reaction on a formalin-fixed, paraffin-embedded tissue is a powerful diagnostic tool, but requires cautious interpretation: a case report

**DOI:** 10.3389/fmed.2026.1736694

**Published:** 2026-02-17

**Authors:** Paddy Ssentongo, Tonya Crook, Rezhan Hussein

**Affiliations:** Division of Infectious Diseases and Epidemiology, Department of Medicine, Penn State Health Milton S. Hershey Medical Center, Hershey, PA, United States

**Keywords:** broad-range polymerase chain reaction, formalin-fixed paraffin-embedded (FFPE) tissue, mediastinal mass or neoplasm, molecular diagnostics, necrotic tissue

## Abstract

A woman in her mid-50s with Lynch syndrome and a history of malignant neoplasms was found to have a superior vena cava (SVC) thrombus and a necrotic mediastinal mass on surveillance imaging. The initial biopsy yielded necrotic debris, and the procedure was complicated by a mediastinal hematoma. Due to concern for possible infection, the patient was empirically treated with antibiotics while planning further workup. A broad-range 16S polymerase chain reaction (BRPCR) was performed on a formalin-fixed paraffin-embedded (FFPE) tissue, which was negative for fungi and mycobacteria but revealed DNA from normal oral flora. Subsequently, she developed SVC syndrome, raising concern about a mediastinal abscess. A repeated biopsy confirmed the suspected carcinoma with necrosis. This case underscores the importance of proper interpretation of BRPCR results in FFPE (non-sterile) tissue and emphasizes the need to use these results in an appropriate clinical context.

## Highlights

- BRPCR on FFPE tissues is vulnerable to contamination and degradation.
- Clinical correlation and appropriate context are essential when ordering or interpreting BRPCR results.
- Fresh tissue collected in a sterile container during the biopsy procedure is preferred for molecular diagnostics when infection is suspected.

## Background

Broad-range 16S polymerase chain reaction (BRPCR) has emerged as a powerful diagnostic tool in culture-negative infections, offering the ability to identify fastidious, slow-growing, or non-viable organisms through amplification of conserved ribosomal gene sequences (1). When used on fresh, sterile tissue, BRPCR can expedite the diagnosis and guide targeted antimicrobial therapy in complex or atypical presentations. Its utility has been documented across a range of clinical scenarios, including prosthetic joint infections, endocarditis, and central nervous system infections, where standard cultures often fail to yield results (2). The technique’s strength lies in its broad scope and sensitivity, allowing the detection of diverse microbial taxa from small or degraded samples, and it has become an increasingly common adjunct in the diagnostic evaluation of suspected infections, particularly when conventional methods are ineffective.

BRPCR amplifies conserved genetic regions, such as the bacterial 16S rRNA gene, the fungal internal transcribed spacer (ITS) regions, and, for acid-fast bacilli, including nontuberculous mycobacteria and the *Mycobacterium tuberculosis* complex, targets such as the 16S rRNA gene, rpoB, and hsp65 to detect a wide range of organisms directly from clinical specimens without the need for culture (3, 4). Sequencing the amplified product and comparing it with reference databases enables organism identification, including fastidious or nonviable pathogens (4).

However, BRPCR has notable limitations, particularly when applied to formalin-fixed paraffin-embedded (FFPE) tissues (5–8). The fixation and embedding process chemically alters nucleic acids, introducing fragmentation and cross-linking that impair amplification fidelity. In necrotic or non-sterile specimens, background DNA from environmental, commensal, or oral flora may be inadvertently amplified, resulting in misleading or false-positive findings. Contamination introduced during tissue processing, DNA extraction, or polymerase chain reaction (PCR) setup can further confound interpretation. These vulnerabilities are especially relevant when BRPCR is ordered on tissue that was not collected for microbiological evaluation and was not handled sterily. The case presented here illustrates how a BRPCR performed on a necrotic FFPE tissue risked misdirecting attention from a non-infectious etiology—an adenocarcinoma masquerading as a mediastinal infection.

## Case presentation

**Figure 1:**
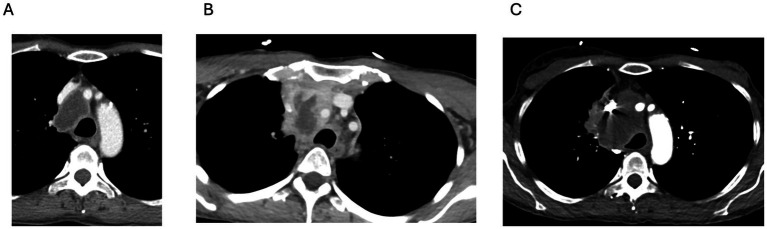
Progressive enlargement of an anterior mediastinal mass over time. **(A)** CT scan of the chest at presentation showing a 4.0 × 2.4 cm centrally necrotic mass in the middle mediastinum with smooth peripheral enhancement and associated SVC involvement and thrombus. **(B)** CT scan of the chest taken approximately 3 weeks later, demonstrating interval enlargement of the lesion with increased peripheral enhancement and a new extension into the anterior mediastinum, involving the left brachiocephalic vein. **(C)** CT scan of the chest taken approximately 8 weeks after the initial presentation, showing further progression with an increase in size to 6.3 × 3.7 cm and possible adjacent lung involvement.

A woman in her mid-50s with Lynch syndrome was found to have a well-circumscribed, 4.0 × 2.4 cm anterior mediastinal mass with central necrosis and peripheral enhancement, which was compressing the proximal superior vena cava (SVC) on routine surveillance computed tomography (CT) of the chest (Figure 1A). She also had an SVC thrombus. Her past medical history included invasive ductal breast carcinoma (diagnosed and treated with mastectomy and adjuvant chemotherapy 9 years prior) and resection of an intestinal neuroendocrine tumor 6 months before presentation. She was anticoagulated and referred to interventional pulmonology for a biopsy and underwent endobronchial ultrasound-guided transbronchial needle aspiration (EBUS-TBNA). Biopsy material was collected for cytologic evaluation and routine cultures. Processing into FFPE blocks occurred according to the routine diagnostic protocol. Pathology from the initial biopsy revealed only necrotic debris with no evidence of malignancy. Twelve days after the EBUS procedure, she developed new neck pain and a bulge along the right side of her neck and presented to the emergency room.

Upon admission, she was afebrile and hemodynamically stable. Laboratory tests showed a white blood cell count of 9.8 × 10^9^/L (normal range: 4.0–10.0 × 10^9^/L), hemoglobin level of 12.3 g/dL (normal range: 12–16 g/dL), platelet count of 198 × 10^9^/L (normal range: 150–400 × 10^9^/L), ESR of 32 mm/h (normal range: 0–15 mm/h), and C-reactive protein level of 2.6 mg/dL (normal range: <0.5 mg/dL). A repeat contrast-enhanced chest CT scan revealed an enlarging necrotic mediastinal mass with mass effect on the SVC (Figure 1B) and left brachiocephalic vein (unchanged from prior), and a new complex, low-attenuation fluid collection measuring up to 4.5 cm in the anterior superior mediastinum, thought to be a hematoma given the recent biopsy. The treating team was concerned about a necrotizing infectious process, prompting an infectious diseases (ID) consultation. The ID examination included blood cultures, urine for Histoplasma and Blastomyces antigens, a serum cryptococcal antigen test, a Quantiferon TB-Gold test, an Aspergillus galactomannan antigen test, Bartonella serology, and BRPCR for fungi, mycobacteria, and bacteria (to look for *Nocardia* species, in particular) was requested to be added to the FFPE specimen as there were no fresh samples to test.

Blood cultures and antigen/serology testing were negative during the hospitalization. However, a computerized tomography Panorex showed a chronic mandibular dental infection, raising concern about the extension of an odontogenic infection via the fascial planes into the mediastinum. The patient was empirically treated with ampicillin-sulbactam IV while hospitalized and discharged on amoxicillin-clavulanate with close outpatient follow-up with pulmonary service. Subsequently, BRPCR was reported to be negative for fungi and mycobacteria but positive for the DNA of *Streptococcus mitis*, *Streptococcus parasanguinis*, *Veillonella atypica*, and *Streptococcus raffinosus*. Since fungi, mycobacteria and nocardia were not detected, she was continued on amoxicillin-clavulanate and advised to undertake further evaluation and proceed with a planned positron emission tomography (PET) scan.

She returned within 8 weeks to the emergency department with progressive dyspnea and worsening right-sided neck swelling. A repeated chest CT scan revealed enlargement of the mediastinal mass (now 6.3 × 3.7 cm) with SVC and inferior vena cava (IVC) invasion (Figure 1C). Heavily influenced by the BRPCR results, the treating teams consulted the ID service again, expressing concern about a mediastinal abscess.

However, the ID service cautioned against a diagnosis of a mediastinal abscess, noting that the BRPCR results could be misleading. They recommended proceeding with a repeat tissue biopsy to obtain a larger sample. A tissue biopsy obtained by CT guidance showed carcinoma with necrosis. Immunostaining was CK7-positive and GATA3/TTF-1/SOX10-negative, indicating a gastrointestinal origin.

## Treatment and follow-up

The patient remained under oncologic care, received chemotherapy, and underwent further evaluation for additional metastatic lesions.

## Discussion

BRPCR has emerged as a useful diagnostic tool for identifying pathogens in culture-negative infections. However, its application to formalin-fixed, paraffin-embedded (FFPE) tissues presents several technical and interpretive challenges. DNA extracted from FFPE specimens is often fragmented and chemically modified due to formalin fixation, reducing amplification fidelity and increasing the likelihood of non-specific or background signals. These limitations are particularly problematic in necrotic or non-sterile tissues, where the distinction between true infection and environmental or commensal contamination becomes blurred (5, 6).

In this case, the detection of DNA from the *Streptococcus mitis* group, *Veillonella atypica*, and other oral flora in a necrotic mediastinal mass led the treating team to consider diagnosing a mediastinal abscess when the patient presented again to the hospital; however, the ID team cautioned against this conclusion and emphasized the importance of repeating a tissue biopsy due to concerns about malignancy. This highlights how molecular results can disproportionately influence decision-making when they are not interpreted within the full clinical context.

Recent literature has reinforced the challenges associated with molecular diagnostics applied to FFPE samples (9–12). Multiple studies highlight that contamination, nucleic acid degradation, and the inability to quantify microbial load substantially limit the clinical specificity of BRPCR in FFPE tissue. Proposed refinements, such as quantitative PCR, metagenomic sequencing, and multiplex molecular panels, may offer improved resolution, although their performance in necrotic or non-sterile tissue remains uncertain (11, 12). These observations underscore the need to interpret FFPE-derived molecular results with caution and always in conjunction with clinical and radiologic data.

A holistic approach that integrates clinical context, radiologic features, histopathology, and microbiology is essential to avoiding premature or inaccurate conclusions (8). Fresh, unfixed, or frozen samples collected in a sterile container, if possible, are preferred for molecular diagnostics to guide antimicrobial therapy, and we urge the cautious interpretation of BRPCR (especially on fixed tissues) if an infectious etiology is not highly suspected, and the use of the results in the proper context.

## Data Availability

The original contributions presented in the study are included in the article/supplementary material, further inquiries can be directed to the corresponding author.

## References

[1] Sontakke S Cadenas MB Maggi RG Diniz PPV Breitschwerdt EB. Use of broad range16S rDNA PCR in clinical microbiology. J Microbiol Methods. (2009) 76:217–25. doi: 10.1016/j.mimet.2008.11.002, 19046999

[2] Akram A Maley M Gosbell I Nguyen T Chavada R. Utility of 16S rRNA PCR performed on clinical specimens in patient management. Int J Infect Dis. (2017) 57:144–9. doi: 10.1016/j.ijid.2017.02.006, 28216180

[3] Rakeman JL Bui U LaFe K Chen Y-C Honeycutt RJ Cookson BT. Multilocus DNA sequence comparisons rapidly identify pathogenic molds. J Clin Microbiol. (2005) 43:3324–33. doi: 10.1128/JCM.43.7.3324-3333.2005, 16000456 PMC1169180

[4] Putnam NE Charles DW Doub JB Johnson JK. Broad-range polymerase chain reaction and sequencing for the diagnosis of infectious diseases. Microbiol Spectr. (2025) 13:e02505-24. doi: 10.1128/spectrum.02505-24, 40042284 PMC11960090

[5] Janda JM Abbott SL. 16S rRNA gene sequencing for bacterial identification in the diagnostic laboratory: pluses, perils, and pitfalls. J Clin Microbiol. (2007) 45:2761–4. doi: 10.1128/JCM.01228-07, 17626177 PMC2045242

[6] Rampini SK Bloemberg GV Keller PM Büchler AC Dollenmaier G Speck RF . Broad-range 16S rRNA gene polymerase chain reaction for diagnosis of culture-negative bacterial infections. Clin Infect Dis. (2011) 53:1245–51. doi: 10.1093/cid/cir692, 21976460

[7] Corless CE Guiver M Borrow R Edwards-Jones V Kaczmarski EB Fox AJ. Contamination and sensitivity issues with a real-time universal 16S rRNA PCR. J Clin Microbiol. (2000) 38:1747–52. doi: 10.1128/JCM.38.5.1747-1752.2000, 10790092 PMC86577

[8] Lehmann U Kreipe H. Real-time PCR analysis of DNA and RNA extracted from formalin-fixed and paraffin-embedded biopsies. Methods. (2001) 25:409–18. doi: 10.1006/meth.2001.1263, 11846610

[9] Fairley JA Gilmour K Walsh K. Making the most of pathological specimens: molecular diagnosis in formalin-fixed, paraffin embedded tissue. Curr Drug Targets. (2012) 13:1475–87. doi: 10.2174/138945012803530125, 22974391

[10] Lam SY Ioannou A Konstanti P Visseren T Doukas M Peppelenbosch MP . Technical challenges regarding the use of formalin-fixed paraffin embedded (FFPE) tissue specimens for the detection of bacterial alterations in colorectal cancer. BMC Microbiol. (2021) 21:297. doi: 10.1186/s12866-021-02359-z, 34715774 PMC8555202

[11] Cruz-Flores R López-Carvallo JA Cáceres-Martínez J Dhar AK. Microbiome analysis from formalin-fixed paraffin-embedded tissues: current challenges and future perspectives. J Microbiol Methods. (2022) 196:106476. doi: 10.1016/j.mimet.2022.106476, 35490989

[12] Hamelin B Hosch S Neidhöfer C Ruf MT Haslbauer JD Field CM . Unbiased DNA pathogen detection in tissues: real-world experience with metagenomic sequencing in pathology. Lab Investig. (2025) 106:104254. doi: 10.1016/j.labinv.2025.104254 41167475

